# Mitochonic acid 5 attenuates age-related neuromuscular dysfunction associated with mitochondrial Ca^2+^ overload in *Caenorhabditis elegans*

**DOI:** 10.1038/s41514-023-00116-2

**Published:** 2023-08-01

**Authors:** XinTong Wu, Miku Seida, Takaaki Abe, Atsushi Higashitani

**Affiliations:** 1 Graduate School of Life Sciences, Tohoku University, Sendai, 980-8577 Japan; 2 Division of Medical Science, Tohoku University Graduate School of Biomedical Engineering, Sendai, 980-0872 Japan; 3 Department of Clinical Biology and Hormonal Regulation, Tohoku University Graduate School of Medicine, Sendai, 980-0872 Japan

**Keywords:** Cell biology, Chemical biology

## Abstract

Mitochonic acid-5 ameliorates the pathophysiology of human mitochondrial-disease fibroblasts and *Caenorhabditis elegans* Duchenne muscular dystrophy and Parkinson’s disease models. Here, we found that 10 μM MA-5 attenuates the age-related decline in motor performance, loss of muscle mitochondria, and degeneration of dopaminergic neurons associated with mitochondrial Ca^2+^ overload in *C. elegans*. These findings suggest that MA-5 may act as an anti-aging agent against a wide range of neuromuscular dysfunctions in metazoans.

Aging in the neuromuscular system includes functional decline, muscle wasting, and weakness, leading to frailty. Central to these aging processes is the accumulation of dysfunctional mitochondria^1,2^. Maintaining normal mitochondrial function is therefore crucial in overcoming the effects of aging. Indole-3-acetic acid (IAA), a plant hormone, is also found in animals, synthesized by liver, kidney^3^ and gut microbes^4,5^. It also accumulates in human renal failure^6^. Additionally, IAA also promotes fibroblast proliferation in both mice and humans^7^. Recent research has even demonstrated that microbiota-derived IAA enhances the effectiveness of chemotherapy in pancreatic ductal adenocarcinoma^8^. Through screening in-house chemical library of IAA derivatives, a compound called Mitochonic Acid 5 (MA-5) was developed. MA-5 exhibits ameliorative effects on fibroblasts from patients with mitochondrial disease^9^. MA-5 enhances ATP production without increasing mitochondrial reactive oxygen species (ROS) generation^9,10^. Furthermore, it has been shown to prolong the survival of a mouse model for mitochondrial disease, known as the “Mitomouse”^11^.

The nematode *C. elegans*, with its relatively short lifespan and molecular similarity to vertebrate systems, offers a valuable model for studying aging. We recently found that MA-5 (final concentration 10 μM) ameliorates the pathogenesis of Duchenne muscular dystrophy (DMD) and Parkinson’s disease (PD) in a nematode model^12^. Thus, one of the next studies aims to investigate whether MA-5 can impede the progression of aging in *C. elegans*.

First, in this study, we found that the administration of 10 μM MA-5 tended to increase endogenous ATP levels in one-day-old young (D1) and mature D4 adults (no significant difference) and markedly ameliorated the age-related decline in ATP levels in D7 and D14 adults (Fig. 1a). At the same time, MA-5 significantly suppressed the age-related decline in locomotor performance as evidenced by higher thrashing rate in liquids and crawling velocity on agar plates compared to the control (Fig. 1b, c). In D1 adults, MA-5 administration also significantly increased thrashing rate, suggesting that MA-5 is a general enhancer of muscle function.

**Fig. 1:**
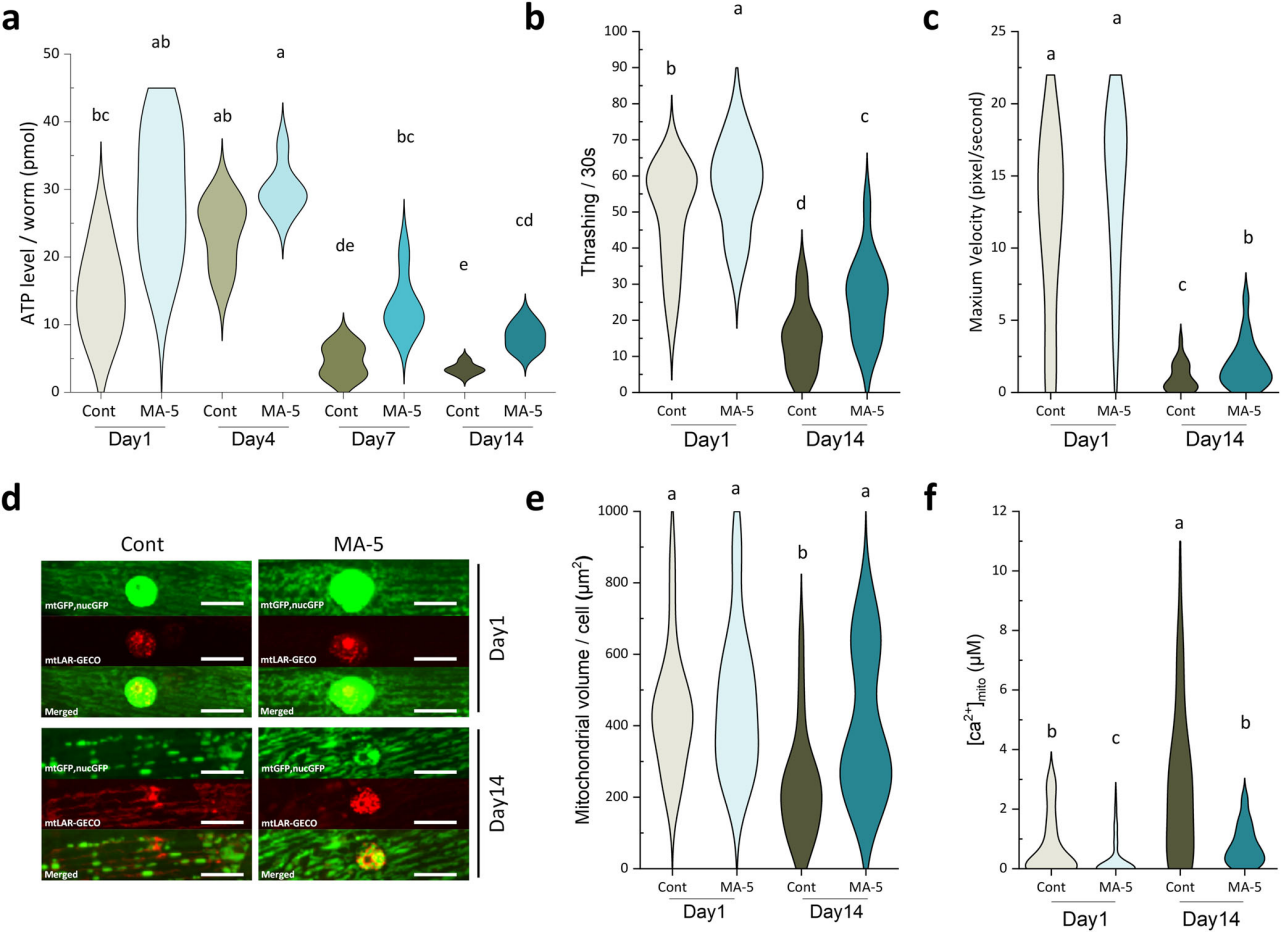
MA-5 improved age-related reduction of motor performance, mitochondrial fragmentation, volume loss, increased [Ca^2+^]mito levels, and decreased ATP levels. **a** ATP levels in ATU3301 (*ccIs4251* [*(pSAK2) myo-3p::GFP::LacZ::NLS* + *(pSAK4)myo-3p::mitochondrialGFP+dpy-20*( + )] *I, acels1* [*myo-3p::mitochondrial LAR-GECO+myo-2p::RFP*] *II* in wild-type N2 background) animals on day 1, 4, 7 and 14 of adulthood cultured with or without MA-5 (*n* = 10–12 worms/treatment). **b** Thrashing rate of D1 and D14 adults of ATU3301 cultured with or without MA-5 was determined in 1 ml M9 for each 30 s (*n* = 28–36 worms/treatment). **c** Maximum velocity of D1 and D14 adults of ATU3301 cultured with or without MA-5 was measured (*n* = 15–35 worms/treatment). **d** Representative images of the mitochondrial morphologies with mtGFP (indicated as green), mitochondrial Ca^2+^ signal with mtLAR-GECO sensor (indicated as red), and merged observed in BWMC of ATU3301 on D1 and D14 adults. Scale bars represent 10 µm. **e** Mitochondria volume in each muscle cell (*n* = 19–24 cells from 5–7 independent worms/treatment) treated with or without MA-5 on day 1 and day 14. **f** Concentration of mitochondrial calcium in BWMC (*n* = 75–141 mitochondria from 6–12 independent worms/treatment). Different letters indicate significant differences (*p* ≤ 0.05) using the Dunn’s test. Data are shown as violin plots. Cont: control treated with 0.1% DMSO; MA-5: 10 μM.

Age-related mitochondria fragmentation and volume loss are known to occur in body wall muscular cells (BWMC), and these impairments correlate well with decreased motor performance^13,14^. Indeed, the administration of MA-5 ameliorated age-related mitochondrial fragmentation and volume loss in elderly D14 (Fig. 1d, e). We recently found that mitochondrial Ca^2+^ ([Ca^2+^]_mito_) levels in BWMC increase with age by using the *aceIs1* transgene of mitochondrial Ca^2+^ sensor mtLAR-GECO (strain ATU3301)^15^. Remarkably, the administration of MA-5 was able to suppress the age-related elevation in [Ca^2+^]_mito_ levels (Fig. 1d, f). Interestingly, MA-5 was also found to maintain low [Ca^2+^]_mito_ levels even in D1 adults (Fig. 1d, f). Furthermore, when compared to the mitochondrial calcium uniporter (MCU) inhibitor Ru360^15^, MA-5 did not inhibit mitochondrial Ca^2+^ oscillations synchronized with cytoplasmic Ca^2+^ oscillations during the muscular contraction and relaxation cycle in *C. elegans* BWMC (Supplementary Fig. 1). This indicates that MA-5 maintains mitochondrial Ca^2+^ homeostasis through an action distinct from MCU inhibition.

To investigate the effects of MA-5 on age-related neurodegeneration, we utilized *vtIs1 dat-1p::*GFP (strain TG2435)^16^ to observe four anterior cephalic dopaminergic neurons (CEPs) that function to sense mechanosensory stimuli^17^. In the mock control, GFP fluorescent puncta increased and became apparent on the dendrites of elderly D16 animals (Fig. 2a, b). These puncta are the formation of axonal spheroids or inclusion bodies commonly observed in degenerating neurons^18^. However, administration of MA-5 significantly reduced the age-related puncta formation (Fig. 2a, b).

**Fig. 2:**
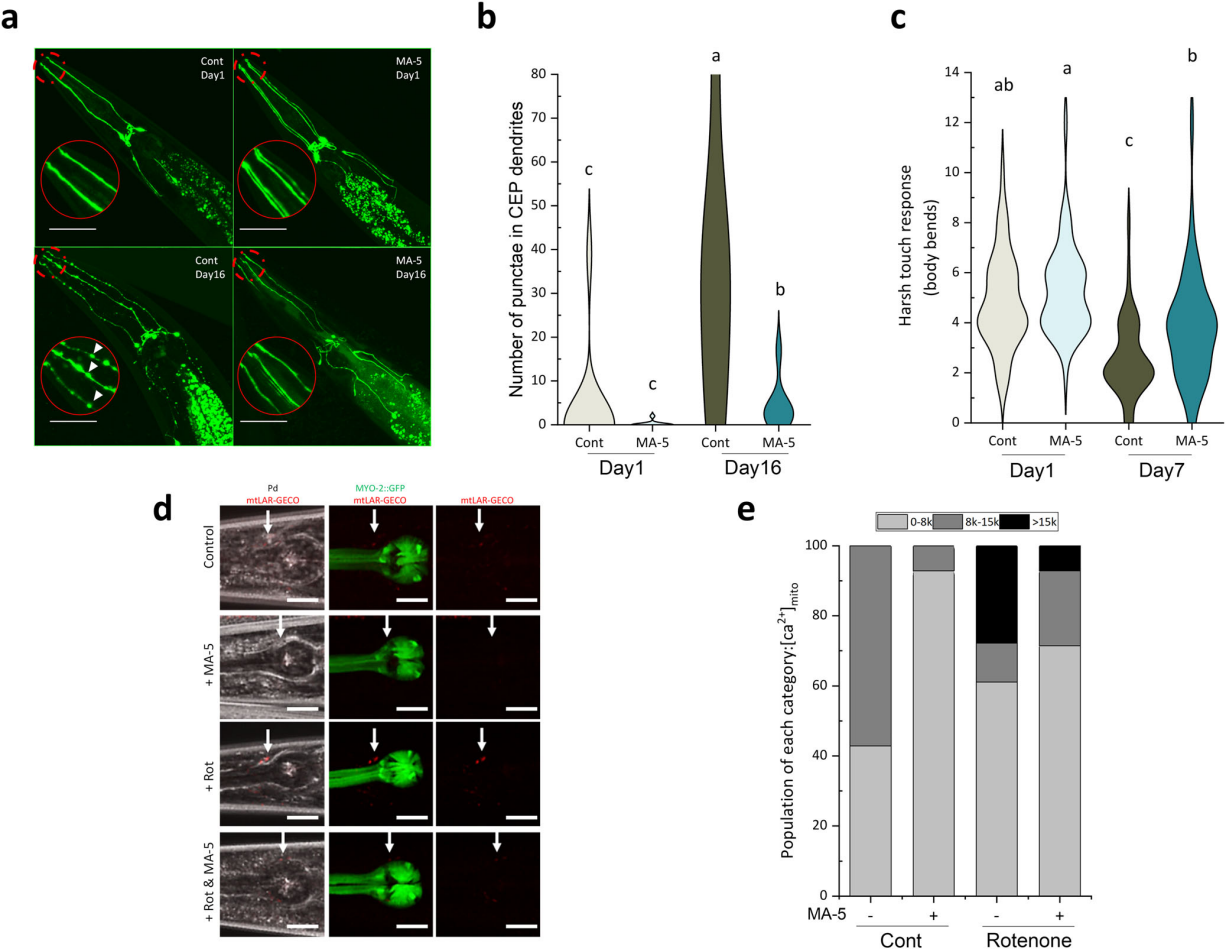
MA-5 suppresses age-related and rotenone-induced neurodegeneration in dopaminergic cephalic (CEP) neurons. **a** Transgenic *C. elegans* TG2435 (*vtIs1* [*dat-1p::GFP + rol-6(su1006)*] V) expressing dopaminergic neurons tagged with GFP cultured with or without MA-5 were monitored on D1 and D16 adults. Representative images of dopamine neuron degeneration with age. (Scale bar: 50 µm). White arrowheads in the enlarged red circles (head part) indicate neuronal processes that exhibit abnormally discontinuous GFP signals. **b** Frequency of the four CEP blebs along the dendrites in animals (*n* = 7–12 worms/treatment). **c** Harsh touch responses of D1 and D7 wild-type N2 adults (*n* = 40 worms/treatment). The response was assayed as the number of reverse body bends a worm makes following a harsh touch stimulus to the head. Data are shown as violin plots. Different letters indicate significant differences (*p* ≤ 0.05) using one-way ANOVA and Tukey’s HSD test. **d** Age-synchronized two-day-old adult worms with *aceIs2* [*dat-1p::mitochondrial LAR-GECO+myo-2p::GFP*] (ATU5301) expressing dopaminergic neurons tagged with mitochondrial Ca^2+^ sensor were monitored following rotenone treatment with or without MA-5 after 24 h. Representative images of [Ca^2+^]_mito_ levels of dopaminergic cephalic (CEP) neurons (Scale bars: 20 µm). White arrowheads in the mitochondrial Ca^2+^ signals of CEPs. **e** Quantitative analysis of mitochondrial Ca^2+^ signals in CEPs (*n* = 7–18 worms in each condition). Z-stack images of mtLAR-GECO fluorescence in CEPs were quantitatively measured by ImageJ software. The fluorescent levels were classified into three categories by A.U. of “0–8k”, “8k–15k”, and “15k≤”. Cont: control treated with 0.1% DMSO; MA-5: 10 μM.

Furthermore, to evaluate the effect of MA-5 on neuronal function decline during aging, harsh touch responses on agar plates^19^ were also observed. In D7 animals, the number of backward body bend responses to head harsh touch decreased by approximately half due to aging, but by about one-third after MA-5 treatment (Fig. 2c). In D16 animals, no backward bending due to harsh touch was observed in either group, but the touch-induced head deflection was observed in half of the control group (*n* = 20/40 worms tested) and in 85% (*n* = 34/40 worms tested) of the MA-5 treated group.

The number of mitochondria in CEPs is much lower than in muscle cells, and age-related changes such as further reduction and fragmentation have not been successfully observed. We therefore constructed and utilized the *aceIs2* [*dat-1p::mitochondrial LAR-GECO+myo-2p::GFP*] transgene (strain ATU5301) in this study to investigate age-related changes in mitochondrial Ca^2+^ levels in CEPs. Based on previous observations, puncta in CEPs increased not only during the aging process but also in young gravid animals 24 h after administration of 2 μM-rotenone, a mitochondrial complex I inhibitor, and these increases were effectively suppressed by MA-5 treatment^12^. Therefore, we aimed to examine whether low-dose rotenone induces an elevation in mitochondrial Ca^2+^ levels in CEPs and whether MA-5 can counteract this effect. The result showed that treatment of ATU5301 D2 animals with 2 μM rotenone for 24 h increased mtLAR-GECO signals in CEPs, which was markedly suppressed by MA-5 administration (Fig. 2d, e). Furthermore, similar to its ability to reduce [Ca^2+^]_mito_ levels in BWMC of D1 and D14 animals (Fig. 1), MA-5 also reduced [Ca^2+^]_mito_ levels in CEPs of D2 animals under control conditions without rotenone treatment. These findings indicate that MA-5 maintains low [Ca^2+^]_mito_ levels not only in muscular cells but also in neurons.

Compared to long-lived *C. elegans* mutants with reduced insulin/insulin-like growth factor-1 signaling (IIS), such as *daf-2* and *age-1* deficiency^20^, MA-5 had no effect on maximum lifespan (Supplementary Fig. 2a). A slight increase in median lifespan was observed, although it was not statistically significant (control: 13.6 ± 0.6 days, MA-5: 15.3 ± 0.5 at 5 μM, 14.8 ± 0.4 at 10 μM and 14.8 ± 0.3 at 20 μM after D1 adults). Additionally, MA-5 at concentrations of 5–20 μM also ameliorated age-related decreases in locomotion (thrashing) in D10 animals and mitochondrial mass in D14 animals (Supplementary Fig. 2b–d). Since MA-5 significantly increased intracellular ATP levels at 3 and 10 µM in a previous study with Hep3B cells^9^, it is likely that similar dose effects are conserved in *C. elegans*. Taken together, MA-5 functions as an anti-aging agent that can significantly improve age-related neuromuscular decline and extend a healthy lifespan.

The suppression of mitochondrial Ca^2+^ overload is a key challenge for ameliorating brain aging and the progress of neurodegenerative diseases such as Alzheimer’s, Parkinson’s and Huntington’s diseases and heart failure^21,22^. Ca^2+^ plays a crucial role in maintaining optimal mitochondrial function. In contrast, an excessive influx of Ca^2+^ can have detrimental effects on mitochondria, resulting in impaired functionality. This overload of Ca^2+^ leads to a decrease in mitochondrial inner membrane potential (ΔΨm) and a reduction in ATP production. Additionally, the increased release of reactive oxygen species (ROS) further exacerbates the damage to mitochondria. Ultimately, these dysfunctions contribute to cellular demise and cell death^23^. Moreover, our recent findings indicate that Ca^2+^ overload promotes mitophagy and results in mitochondrial volume loss in *C. elegans* BWMC^15^. Both genetic (*mcu-1* mutation) and pharmacological (Ru360 administration) suppression of MCU can ameliorate muscle weakness induced by *C. elegans* aging and *dys-1 (eg33)* mutation of DMD model^15^. However, MA-5 administration reduced mitochondrial Ca^2+^ overload induced by aging, rotenone treatment (Figs. 1, 2), and the *dys-1 (eg33)* mutation^12^, while maintaining MCU activity. These results highlight the role of MA-5 in maintaining mitochondrial homeostasis by inhibiting excessive Ca^2+^ accumulation, rather than inhibiting normal Ca^2+^ uptake into mitochondria.

MA-5 stabilizes mitochondrial cristae structures through binding to Mitofilin/Mic60 in cultured mammalian cells and facilitates ATP synthetase oligomerization^10^. Here we also examined whether Mitofilin/Mic60 is the target of MA-5 in *C. elegans* as in mammalian cells. Our previous data showed that MA-5 fluorescently labeled with BODIPY efficiently translocated into mitochondria of live wild-type *C. elegans*^12^. Using this experimental system, BODIPY-MA-5 signal was markedly reduced in mitochondria of gonadal and oocyte cells of deletion mutants of each of the two Mitofilin genes *immt-1* and *immt-2*^24^ in *C. elegans* genome (Supplementary Fig. 3). In the *immt-1* and *immt-2* deletion double mutants, the mitochondrial fluorescence signal of BODIPY-MA-5 was almost completely lost (Supplementary Fig. 3). These results strongly suggest that MA-5 binds in common with Mitofilin/Mic60/IMMT-1 and IMMT-2, which are conserved in metazoans. MA-5 also improves distended cristae in *C. elegans immt-1* single mutants^12^. Mitofilin/Mic60 depletion led to a loss of cristae junctions (CJs)^25^. Intriguingly, MICU-1, one of the components of the MCU complex, was also recently reported to be important not only for Ca^2+^ transport but also for maintenance of CJs width^26^. MICU-1 depletion widened the CJs, increased the release of cytochrome *c*, and loss of ΔΨm^26^. Overall, our study suggests that stabilization of mitochondrial CJs by MA-5 causes not only enhanced ATP production but also maintenance of mitochondrial Ca^2+^ homeostasis. This homeostatic effect of MA-5 maintains mitochondrial quality and extends a healthy life span.

## Methods

### *C. elegans* strains and culture conditions

The strains used in this study are as follows: wild-type N2, ATU2301: *goeIs3* [*myo-3p::SL1::GCamP3.35::SL2::unc54 3’UTR+unc-119(*+*)*] *V*^15^*, acels1*[*myo-3p::mitochondrial LAR-GECO+myo-2p::RFP*] *II*, ATU3301: *ccIs4251* [*(pSAK2) myo-3p::GFP::LacZ::NLS* + *(pSAK4)myo-3p::mitochondrialGFP+dpy-20*+*)*] *I, acels1* [*myo-3p::mitochondrial LAR-GECO+myo-2p::RFP*] *II*^15^, ATU3307: *ccIs4251, aceIs1, immt-1* (*tm1730*), ATU3308: *ccIs4251, aceIs1, immt-2* (*tm2366*), ATU3310: *ccIs4251, aceIs1, immt-1* (*tm1730*), *immt-2* (*tm2366*), TG2435: *vtIs1* [*dat-1p::GFP + rol-6(su1006)*] *V*, and ATU5301: *aceIs2* [*dat-1p::mitochondrial LAR-GECO+myo-2p::GFP*]. The nematodes were synchronously cultured from the eggs on *Escherichia coli* OP50 NGM agar plates (60 mm diameter, 8 ml volume) at 20 °C. MA-5 (Hayashi K-I, Okayama University of Science) and the ETC inhibitor rotenone (Millipore Sigma, Burlington, MA, USA) were applied to the OP50 seeded plates at a final concentration of 5, 10, 20, and 2 μM, respectively. These plates were allowed to permeabilize for 24 h and used for nematode culture.

### ATP detection

ATU3301 worms on desired days were collected in 100 µM M9 buffer for further ATP assays. Endogenous ATP was extracted by 3 cycles of sonication (15”, 60” resting at 20 kHz, Ultrasonic Homogenizer Smurt NR-50M, Microtec Co. Ltd, Cheshire, CT, USA) and centrifugation for 1 min at 5000 g. An ATP determination kit (Molecular Probes, Eugene, OR, USA) was used to measure endogenous ATP levels.

### Lifespan and motor activity analyses

A total of 300 worms at the L4 stage were set up on three replicate solid media with or without MA-5 treatment under 20 °C. Worms were gently touched with a worm picker to record the number of worms alive, dead, or censored for each day. The Kaplan–Meier survival curves were performed using Microsoft Excel. To analyze the motor activity, the thrashing frequency of synchronized adult worms was measured in 1 ml of M9 buffer for 30 s. The maximum velocity was determined by transferring ATU3301 animals to new NGM-agar plates without bacterial lawn, irradiating them with blue light (GFP-B mode: Excitation wavelength 480 nm and Emission bandwidth 40 nm) using a fluorescence stereomicroscope (SMZ18; Nikon, Tokyo, Japan), video recording their movement behavior using a microscope camera (DP74; Olympus, Tokyo, Japan), and calculating by ImageJ software.

### Mitochondria and mitochondrial Ca^2+^ levels measurement

*C. elegans* BWMC and their mitochondrial images were obtained using confocal laser-scanning microscopy (FluoView Olympus FV10i; Olympus, Tokyo, Japan). Synchronized worms were washed with M9 buffer, mounted on a microscope slide (6.5-mm square, 20-μm deep well made with a water-repellent coating (Matsunami Glass Ind., Ltd. Osaka, Japan) with 100 mM NaN3 solution, and immediately observed. Muscular mitochondrial volume and length of mitochondrial networks were analyzed by Image J software (National Institutes of Health, Bethesda, MD, USA). For live imaging of the cytoplasmic and mitochondrial Ca^2+^ oscillation in BWMC using GCaMP fluorescence (*goeIs3* transgene) and mtLAR-GECO (*aceIs1* transgene), the synchronized ATU2301 worms were washed and mounted with 2.5% polystyrene microspheres (0.10 μm, Polysciences Inc. Warrington, PA, USA). The [Ca^2+^]_mito_ was calculated using the following equation: [Ca^2+^]_mito_ = K_d_ ∙ (R − R_min_) / (R_max_ − R), where K_d_ (12 μM) indicates the dissociation constant between Ca^2+^ and the LAR-GECO probe, and R indicates the ratio of fluorescence intensity of mtLAR-GECO to that of mtGFP^15^. Time-lapse confocal images of cytosolic GCaMP fluorescence were acquired at room temperature (20~22 °C) by FV10i. In dopaminergic cephalic (CEP) neurons, mitochondrial Ca^2+^ levels were monitored by the mitoLAR-GECO fluorescent intensities of ATU5301 carrying *aceIs2* [*dat-1p::mitochondrial LAR-GECO+myo-2p::GFP*]. Day 1 adults of ATU5301 were treated with MA-5 and rotenone for 24 h and the mitoLAR-GECO signal levels were observed and measured by Fv10i z-stack images.

### Dopaminergic neuronal degeneration measurement

Age-synchronized adult day 1 and day 16 worms with *dat-1p::GFP* (TG2435) were used in this experiment. Approximately 12 worms were analyzed for each condition. Images were obtained using confocal laser-scanning microscopy, and ImageJ software was used to calculate the number of beads in all four CEP neurons.

### Harsh touch response

A total of 40 wild-type N2 (each D1, D7, and D16 synchronized adults) were analyzed under each experimental condition. The head of the forward-moving worm was touched with a platinum wire, and the number of backward body bends was counted using a stereomicroscope (SZ61; Olympus, Tokyo, Japan)^19^.

### BODIPY-based fluorescent-conjugated MA-5 staining

ATU3301 and its derivatives with *immt-1* and *immt-2* deletion mutants were stained with 2 μM BODIPY-MA-5^11^ for 2 h. After washing with M9 buffer and fixing with 100 mM NaN_3_, the fluorescent images of BODIPY-MA-5 were immediately observed using a confocal laser-scanning microscope (Olympus, Tokyo, Japan) at a constant laser power of Ex 490 / Em 504 nm.

### Statistical analysis

The one-way ANOVA with post-hoc Tukey’s HSD and Dunn’s test were used for comparisons between groups as appropriate (R or Origin software). All data points including outliers were used for means and statistical significance. A *p*-value of <0.05 was considered significant. Different letters indicate significant differences between the groups.

### Reporting summary

Further information on research design is available in the Nature Research Reporting Summary linked to this article.

## Supplementary information

Supplementary Figures

Reporting summary

## Data Availability

Data sets generated from this study are available from the corresponding author upon reasonable request.
