# Supplementary material for: Mitochonic acid 5 attenuates age-related neuromuscular dysfunction associated with mitochondrial Ca2+ overload in Caenorhabditis elegans
Source: NPJ Aging. 2023 Aug 1;9(1):20. doi: 10.1038/s41514-023-00116-2 (PMC10394014; doi:10.1038/s41514-023-00116-2)
Supplement: Supplementary file 1 — Supplementary Figures [file 41514_2023_116_MOESM1_ESM.pdf]

Supplementary Figure 1

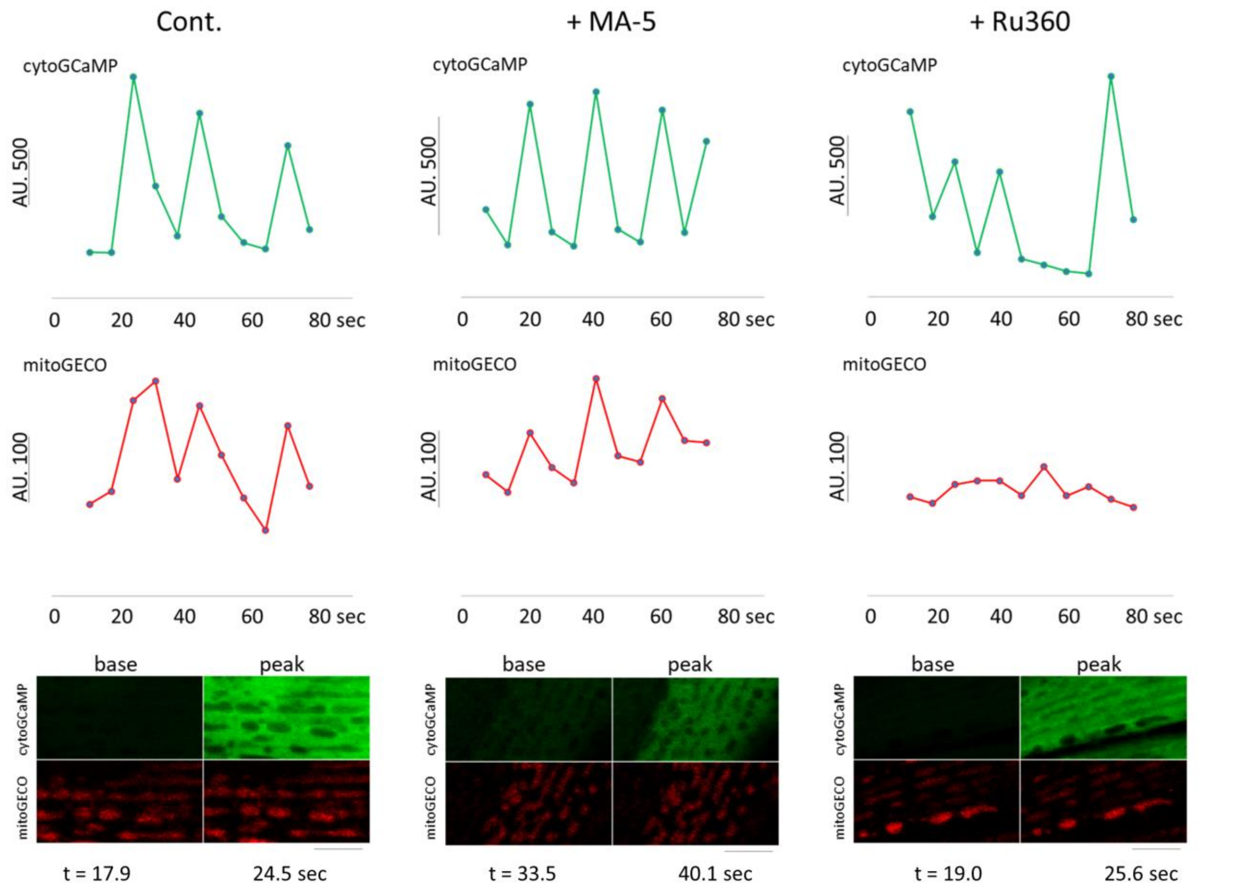

Supplementary Figure 1

Effect of MA-5 on cytosolic and mitochondrial Ca^{2+} levels fluctuated in synchronization with contraction and relaxation of BWMC. Time-lapse confocal images of cytosolic GCaMP and mtGECO fluorescence in BWMC of ATU2301 D2 adults were acquired at room temperature (20 ~ 22 ºC) by an FV10i confocal laser-scanning microscope (Olympus). Typical fluorescent images of muscle cytosolic GCaMP and mtGECO at low and high $[Ca^{2+}]_{cyto}$ levels during muscular contraction and relaxation were shown in the bottom panel. Mitochondrial Ca^{2+} uptake synchronized with cytosolic Ca^{2+} upregulation was suppressed by the administration of MCU-1 inhibitor Ru360 but not MA-5 treatment.

Supplementary Figure 2

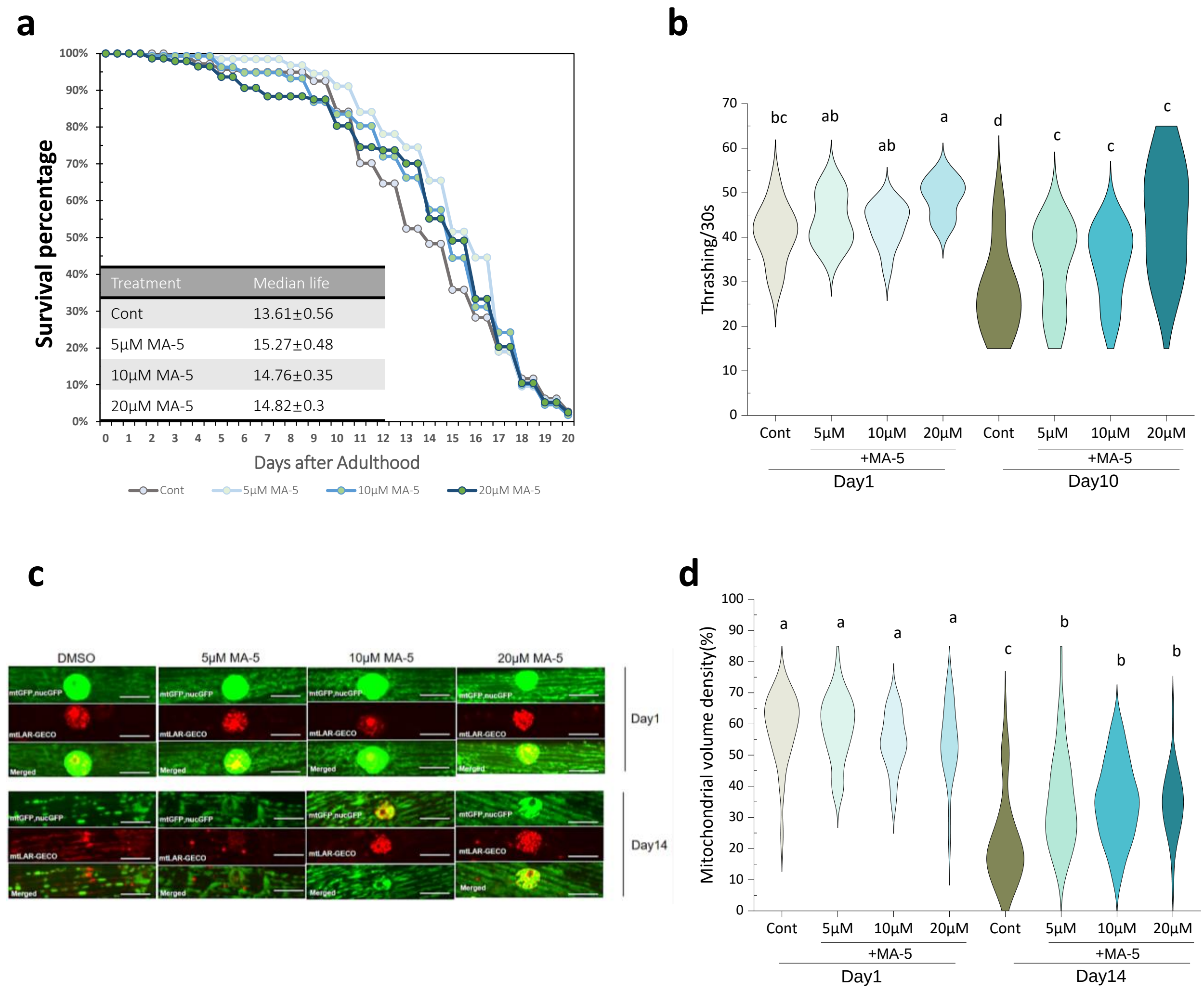

Supplementary Figure 2

Effect of MA-5 on *C. elegans* lifespan, age-related reduction of motor performance, mitochondrial fragmentation and volume loss. (a) Lifespan experiments with different concentrations of MA-5. MA-5 did not significantly prolong the maximum lifespan at 5-20 µM, but a little (not significant) extended the median life span by approximately one day (control: 13.6 ± 0.6 days, MA-5: 15.3 ± 0.5 at 5 µM, 14.8 ± 0.4 at 10 µM and 14.8 ± 0.3 at 20 µM after D1 adults). Strain ATU3301 was used (n = 150 each condition). **(b)** Thrashing rate of D1 and D10 adults of ATU3301 cultured with or without MA-5 was determined in 1 ml M9 for each 30 seconds (n = 10 worms/treatment). **(c)** Representative images of the mitochondrial morphologies with mtGFP (indicated as green), mitochondrial Ca^{2+} signal with mtLAR-GECO sensor (indicated as red), and merged observed in BWMC of ATU3301 on D1 and D14 adults. Scale bars represent 10 µm. **(d)** Mitochondrial volume in each muscle cell (percentile of Z-stack image) (n = 26–68 cells from 5–7 independent worms/treatment) treated with or without MA-5 on day 1 and day 14. Different letters indicate significant differences ($p \leq 0.05$) using the Dunn's test with all data points including outliers. Data are shown as violin plots. Cont: control treated with 0.1% DMSO; MA-5: 5, 10, and 20 µM.

Supplementary Figure 3

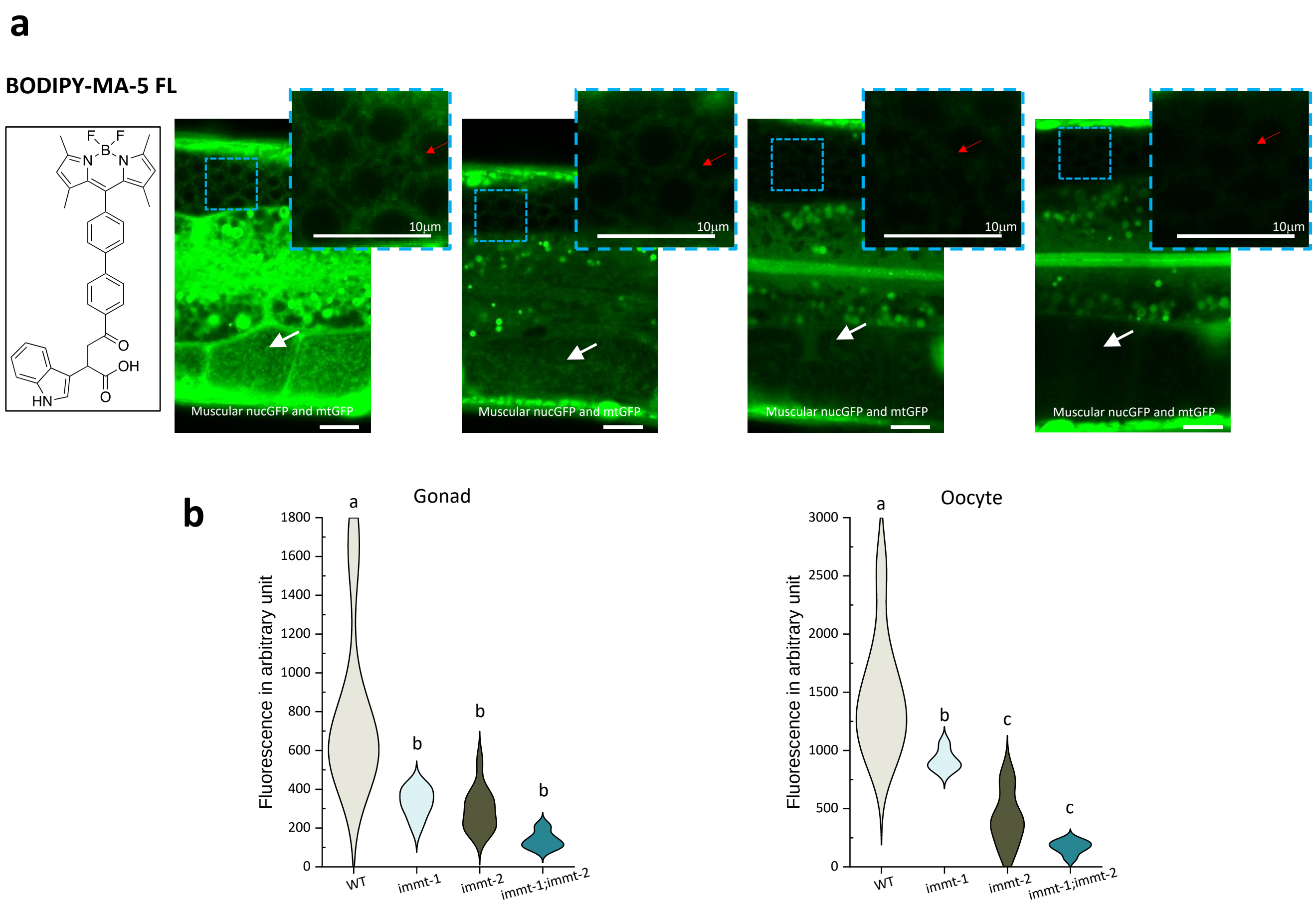

Supplementary Figure 3

MA-5 interacts with the Mitofilins encoded by *immt-1* and *immt-2* genes in *C. elegans*.

(a) Fluorescent images of BODIPY-MA-5 on mitochondria are indicated in gonadal germ cells (enlarged square with red arrows) and mature oocytes intestine (white arrows) of ATU3301 (wild type), ATU3307 *immt-1* (*tm1730*), ATU3308 *immt-2* (*tm2366*), and ATU3310 *immt-1* (*tm1730*), *immt-2* (*tm2366*). The images of each D2 adult after 2 hours of staining with 2 µM BODIPY-MA-5 were monitored by confocal microscopy. Fluorescent excitation and emission wavelengths (nm) were under 490/504. Scale bars represent 10 µm. **(b)** Quantitative analysis of BODIPY-MA-5 signals in mitochondria of gonadal and oocyte cells (*n* = 12–18 mitochondria from 4–6 independent worms in each condition). Signal levels (A.U.) were measured quantitatively with FV10i-ASW ver. 4.2 software (Olympus, Tokyo, Japan).
