# Supplementary material for: Mitochonic acid 5 attenuates age-related neuromuscular dysfunction associated with mitochondrial Ca2+ overload in Caenorhabditis elegans
Source: NPJ Aging. 2023 Aug 1;9(1):20. doi: 10.1038/s41514-023-00116-2 (PMC10394014; doi:10.1038/s41514-023-00116-2)
Supplement: Supplementary file 2 — Reporting summary [file 41514_2023_116_MOESM2_ESM.pdf]

nature portfolio

Corresponding author(s): Atsushi Higashitani

Last updated by author(s): May 31, 2023

Reporting Summary

Nature Portfolio wishes to improve the reproducibility of the work that we publish. This form provides structure for consistency and transparency in reporting. For further information on Nature Portfolio policies, see our Editorial Policies and the Editorial Policy Checklist.

Statistics

For all statistical analyses, confirm that the following items are present in the figure legend, table legend, main text, or Methods section.

n/a	Confirmed	
☐	☒	The exact sample size (*n*) for each experimental group/condition, given as a discrete number and unit of measurement
☐	☒	A statement on whether measurements were taken from distinct samples or whether the same sample was measured repeatedly
☐	☒	The statistical test(s) used AND whether they are one- or two-sided<br>*Only common tests should be described solely by name; describe more complex techniques in the Methods section.*
☐	☒	A description of all covariates tested
☐	☒	A description of any assumptions or corrections, such as tests of normality and adjustment for multiple comparisons
☐	☒	A full description of the statistical parameters including central tendency (e.g. means) or other basic estimates (e.g. regression coefficient) AND variation (e.g. standard deviation) or associated estimates of uncertainty (e.g. confidence intervals)
☐	☒	For null hypothesis testing, the test statistic (e.g. F, t, r) with confidence intervals, effect sizes, degrees of freedom and P value noted<br>*Give P values as exact values whenever suitable.*
☒	☐	For Bayesian analysis, information on the choice of priors and Markov chain Monte Carlo settings
☒	☐	For hierarchical and complex designs, identification of the appropriate level for tests and full reporting of outcomes
☒	☐	Estimates of effect sizes (e.g. Cohen's d, Pearson's r), indicating how they were calculated

Our web collection on statistics for biologists contains articles on many of the points above.

Software and code

Policy information about availability of computer code

Data collection	MS Excel, Origin
Data analysis	Dunn's test of multiple comparisons using Rank Sums, One-way ANOVA and Tukey's HSD test,

For manuscripts utilizing custom algorithms or software that are central to the research but not yet described in published literature, software must be made available to editors and reviewers. We strongly encourage code deposition in a community repository (e.g. GitHub). See the Nature Portfolio guidelines for submitting code & software for further information.

Data

Policy information about availability of data

All manuscripts must include a data availability statement. This statement should provide the following information, where applicable:

- Accession codes, unique identifiers, or web links for publicly available datasets
- A description of any restrictions on data availability
- For clinical datasets or third party data, please ensure that the statement adheres to our policy

Data sets generated from this study are available from the corresponding author upon reasonable request.

Research involving human participants, their data, or biological material

Policy information about studies with human participants or human data. See also policy information about sex, gender (identity/presentation), and sexual orientation and race, ethnicity and racism.

Reporting on sex and gender	n/a
Reporting on race, ethnicity, or other socially relevant groupings	n/a
Population characteristics	n/a
Recruitment	n/a
Ethics oversight	n/a

Note that full information on the approval of the study protocol must also be provided in the manuscript.

Field-specific reporting

Please select the one below that is the best fit for your research. If you are not sure, read the appropriate sections before making your selection.

☒ Life sciences ☐ Behavioural & social sciences ☐ Ecological, evolutionary & environmental sciences

For a reference copy of the document with all sections, see nature.com/documents/nr-reporting-summary-flat.pdf

Life sciences study design

All studies must disclose on these points even when the disclosure is negative.

Sample size	Based on reviewed previous studies in the field and conducted pilot experiments to estimate the expected effect size. These preliminary investigations allowed us to determine a reasonable estimate of the magnitude of the difference we anticipated observing between the experimental groups.we set the level of statistical significance at α = 0.05, which is a commonly accepted threshold in scientific research.Moreover, we planned to utilize a one-way ANOVA with post-hoc Tukey's HSD and Dunn's test, which is commonly employed for analyzing the type of data collected in our study.
Data exclusions	No data were excluded from the analyses
Replication	All attempts at replication were successful.We took several measures to verify the reproducibility of our experimental findings, including detailed documentation of protocols, adherence to standardized methodologies, independent replications and robust statistical analyses.
Randomization	Sample allocation into experimental groups was performed using a randomization process. We employed a simple randomization method and matched worms based on age and developmental stage to ensure uniformity across the groups.
Blinding	The investigator were blinded to group allocation during data collection and/or analysis.

Reporting for specific materials, systems and methods

We require information from authors about some types of materials, experimental systems and methods used in many studies. Here, indicate whether each material, system or method listed is relevant to your study. If you are not sure if a list item applies to your research, read the appropriate section before selecting a response.

Materials & experimental systems

n/a	Involved in the study	
☒	☐	Antibodies
☒	☐	Eukaryotic cell lines
☒	☐	Palaeontology and archaeology
☐	☒	Animals and other organisms
☒	☐	Clinical data
☒	☐	Dual use research of concern
☒	☐	Plants

Methods

n/a	Involved in the study	
☒	☐	ChIP-seq
☒	☐	Flow cytometry
☒	☐	MRI-based neuroimaging

Animals and other research organisms

Policy information about studies involving animals; ARRIVE guidelines recommended for reporting animal research, and Sex and Gender in Research

Laboratory animals	Caenorhabditis elegans:wild-type N2 at day1, day7 and day16 adults ,<br>ATU2301: goeIs3 [myo-3p::SL1::GCamP3.35::SL2::unc54 3'UTR + unc-119(+)] V 15, aceIs1[myo-3p::mitochondrial LAR-GECO +myo-2p::RFP] II at day2 adults,<br>ATU3301: ccIs4251 [(pSAK2) myo-3p::GFP::LacZ::NLS+(pSAK4)myo-3p::mitochondrialGFP+dpy-20(+)] I, aceIs1 [myo-3p::mitochondrial LAR-GECO+myo-2p::RFP] II 15 at day1, day10 and day14 adults,<br>ATU3307: ccIs4251, aceIs1, immt-1 (tm1730) at day2 adults,<br>ATU3308: ccIs4251, aceIs1, immt-2 (tm2366) at day2 adults,<br>ATU3310: ccIs4251, aceIs1, immt-1 (tm1730), immt-2 (tm2366) at day2 adults,<br>TG2435: vtIs1 [dat-1p::GFP + rol-6(su1006)] V at day1 and day16 adults,<br>and ATU5301: aceIs2 [dat-1p::mitochondrial LAR-GECO+myo-2p::GFP] at day1 adults.
Wild animals	n/a
Reporting on sex	n/a
Field-collected samples	n/a
Ethics oversight	C. elegans strains were provided by the Caenorhabditis Genetics Center funded by the U.S. National Institutes of Health (NIH) Office of Research Infrastructure Program (P40 OD010440) and the National Bioresource Project, Tokyo, Japan.

Note that full information on the approval of the study protocol must also be provided in the manuscript.
